# Serum Malondialdehyde is Associated with Non-Alcoholic Fatty Liver and Related Liver Damage Differentially in Men and Women

**DOI:** 10.3390/antiox9070578

**Published:** 2020-07-02

**Authors:** Shira Zelber-Sagi, Dana Ivancovsky-Wajcman, Naomi Fliss-Isakov, Michal Hahn, Muriel Webb, Oren Shibolet, Revital Kariv, Oren Tirosh

**Affiliations:** 1 School of Public Health, University of Haifa, Haifa 3498838, Israel; divancov@campus.haifa.ac.il; 2 Department of Gastroenterology, Tel Aviv Medical Center, Tel Aviv 6423914, Israel; naomifl@tlvmc.gov.il (N.F.-I.); murielw@tlvmc.gov.il (M.W.); orensh@tlvmc.gov.il (O.S.); revitalk@tlvmc.gov.il (R.K.); 3 Sackler Faculty of Medicine, Tel Aviv University, Tel Aviv 6997801, Israel; 4 Institute of Biochemistry, Food Science and Nutrition, The RH Smit Faculty of Agriculture, Food and Environment, The Hebrew University of Jerusalem, Rechovot 76100001, Israel; Michalhahn@pac.ac.il (M.H.); oren.tirosh@mail.huji.ac.il (O.T.)

**Keywords:** fatty liver, lipid peroxidation, antioxidants, oxidative stress

## Abstract

Background: Non-alcoholic fatty liver disease (NAFLD) and steatohepatitis (NASH) are associated with increased oxidative stress and lipid peroxidation, but large studies are lacking. The aim was to test the association of malondialdehyde (MDA), as a marker of oxidative damage of lipids, with NAFLD and liver damage markers, and to test the association between dietary vitamins E and C intake and MDA levels. Methods: A cross-sectional study was carried out among subjects who underwent blood tests including FibroMax for non-invasive assessment of NASH and fibrosis. MDA was evaluated by reaction with Thiobarbituric acid and HPLC-fluorescence detection method. NAFLD was diagnosed by abdominal ultrasound. Findings: MDA measurements were available for 394 subjects. In multivariate analysis, the odds for NAFLD were higher with the rise of MDA levels in a dose–response manner, adjusting for age, gender, BMI, and lifestyle factors. Only among men, higher serum MDA was associated of higher odds for NAFLD and NASH and/or fibrosis (OR = 2.59, 95% CI 1.33–5.07, *P* = 0.005; OR = 2.04, 1.02–4.06, *P* = 0.043, respectively). Higher vitamin E intake was associated with lower odds of high serum MDA level (OR = 0.28 95% CI 0.13–0.62, *P* = 0.002). In conclusion, serum MDA is associated with NAFLD and markers of NASH or fibrosis among men. Dietary vitamin E may be protective among women.

## 1. Introduction

Nonalcoholic fatty liver disease (NAFLD) is emerging as the most common chronic liver condition worldwide, with global prevalence in the general population estimated to be 25% [1]. NAFLD includes two pathologically distinct conditions: Non-alcoholic fatty liver which is simple steatosis, and non-alcoholic steatohepatitis (NASH) which can progress to liver fibrosis, cirrhosis, and hepatocellular carcinoma (HCC); both conditions are associated with extrahepatic manifestations such as cardiovascular disease [1,2,3]. Oxidative stress is involved in NAFLD pathogenesis and especially in the onset and progression NASH [4]. Patients with NASH have increased oxidative stress, lipid peroxidation, and inflammation, paralleled with reduced antioxidant defense capacities [5,6]. Extensive research has identified oxidative stress-induced inflammation with lipid peroxidation, cytokine activation, as well as excess production of reactive oxygen and nitrogen species (ROS/RNS) as possible mechanisms in NASH pathogenesis [7]. During the process of lipid peroxidation, a wide range of pre-inflammatory products are produced which result in progression of the disease. One of these by-products is malondialdehyde (MDA), which is a common marker for oxidative stress. Furthermore, MDA can stimulate hepatic stellate cells to produce collagen which results in fibrosis [8]. An increase in serum oxidative markers (e.g., MDA) paralleled by a decrease in the activity of antioxidants has been observed in patients with NAFLD [9]. Plasma MDA levels were demonstrated to be significantly increased in diabetic or obese NAFLD patients as compared with healthy controls [10]. Moreover, anti-MDA antibodies in 167 biopsy proven NAFLD patients were demonstrated to be associated with higher risk of having advanced fibrosis, but not with necroinflammation [11]. Interestingly, patients with NAFLD were demonstrated to have significantly higher levels of MDA and other oxidative markers in comparison to chronic viral hepatitis patients [12].

Dietary antioxidants could be an effective strategy to prevent NASH. Vitamin E and vitamin C are two major antioxidants, which are fat-soluble and water-soluble, respectively. Vitamin E plays a role in anti-inflammatory activities, gene expression, cellular signaling, and cell proliferation [13]. Vitamin E encompasses a group of 8 plant based molecules; the most abundant form is α-tocopherol [14]. Due to its ability to inhibit ROS production during the development of steatohepatitis, vitamin E has been investigated as a therapeutic agent in NAFLD and especially NASH [15]. In experimental studies, vitamin E supplement reduced oxidative stress [16], serum transaminase levels and hepatic steatosis in mouse NASH model [17]. The association between vitamin C dietary intake and NAFLD has been studied, but not extensively, and with conflicting results [18,19,20,21].

To our best knowledge, there are no large studies that tested the association of oxidative damage of lipids with human NAFLD and NASH, thoroughly adjusting for metabolic and lifestyle risk factors and evaluating the potential protective role of dietary vitamin E and C. Therefore, the aim of the current study was first, to test the association of MDA, as a marker of oxidative damage of lipids, with NAFLD and liver damage markers of NASH and fibrosis, and second, to test the association between dietary vitamins E and C intake and MDA levels, in a cohort of subjects from general population. We hypothesized that MDA will be related with increased hepatic damage and that dietary intake of these antioxidants will be protective from oxidative damage of lipids.

## 2. Materials and Methods

### 2.1. Study Design and Population

A cross sectional study among 40–70-year-old subjects who underwent screening colonoscopy at the Department of Gastroenterology and Hepatology in the Tel-Aviv Medical Center, and agreed to participate in a metabolic and hepatic screening study between the years 2010 and 2015. Exclusion criteria included: Presence of HBsAg or anti-HCV antibodies, fatty liver suspected to be secondary to hepatotoxic drugs, and excessive alcohol consumption (≥30 g/day in men or ≥20 g/day in women). In addition, subjects who reported an unreasonable caloric intake were excluded; below or above the acceptable range for men 800–4000 Kcal/day and for women 500–3500 Kcal/day [22].

The study was approved by the Tel-Aviv medical center IRB committee and all participants signed an informed consent.

### 2.2. Data Collection and Definition of Hepatic and Metabolic Variables

Study participants were invited for a single day visit, in which they underwent fasting blood tests, liver ultrasound, a face-to-face interview using a structured questionnaire, assembled by the Israeli Ministry of Health and used in national surveys [23], including demographic details, health status, alcohol consumption, smoking and exercise habits. In addition, they completed food frequency questionnaire (FFQ). To avoid report bias, the participants were informed of their abdominal ultrasonography (AUS) and blood tests results only after completing the questionnaires. Fatty liver was diagnosed by AUS using standardized criteria [24] as previously described [25], performed in all subjects with the same equipment (EUB-8500 scanner Hitachi Medical Corporation, Tokyo, Japan) and by the same experienced radiologist (Webb M).

Presumed NASH and fibrosis were evaluated non-invasively by FibroMax which includes FibroTest, new quantitative NashTest, and SteatoTest, (BioPredictive, Paris, France), and has been validated extensively [26,27]. The FibroTest includes serum α2-macroglobulin, apolipoprotein-A1, haptoglobin, total bilirubin, and γ-glutamyl transpeptidase adjusted for age and gender. The NashTest 2 includes all above and alanine aminotransferase, serum cholesterol, and triglycerides [27]. The presence of fibrosis was defined as ≥F2, corresponding to a value ≥0.48, indicating significant fibrosis [26]. The presence of presumed NASH was defined as ≥N2, corresponding to a value ≥0.50 [26]. The procedures were those recommended by BioPredictive, including exclusion of non-reliable results [28].

Type-2 diabetes or pre-diabetes were defined as fasting glucose ≥126 or 100 mg/dL and/or HbA1C ≥6.5% or 5.7% (respectively) and/or use of diabetic medications.

### 2.3. Nutritional and Lifestyle Variables Evaluation and Definitions

The semi-quantitative FFQ, which was assembled by the Food and Nutrition Administration, Ministry of Health and tailored to the Israeli population, is composed of 117 food items with specified serving sizes. For each food item, participants indicated their average frequency of consumption over the past year. The nutrient components were obtained from the Israeli National Nutrient Database (BINAT), Ministry of Health. Vitamin intake was calculated as mg/day. Since subjects with high vitamin intake also consume more calories, we corrected vitamin consumption per 1000 Kcal. High intake of vitamins was defined as above the study upper tertile (corresponding to for vitamin E ≥8.43, 8.40, 8.48 mg/1000 Kcal for total population, women or men; for vitamin C ≥97.83, 102.84, 94.93 mg/1000 Kcal for total population, women or men, respectively).

### 2.4. Determination of Malondialdehyde Serum Levels

Malondialdehyde was extracted from serum samples by treatment with 20 percent TCA at ration of 1:1. The serum samples were centrifuge at 8000 rpm and filtered through a 0.2 μm membrane. The supernatant was treated Thiobarbituric acid (20 mM) and boiled in a water bath for 60 min. A 10 μL sample was injected into an high-performance liquid chromatography (HPLC) (Merck Hitachi) and separated with a C-18 phanomenex column, model RP-18, and detected with an HPLC fluorescence detector (Shimadzu, Japan) set at 532 nm excitation and 553 nm emission. The mobile phase consisted of a 35:65 (*v*/*v*) mixture of methanol and 0.05 M potassium phosphate buffer, pH 7, and the flow rate was 1 mL/min. MDA standard solutions were used to generate a standard curve. The nM levels of MDA found by us in the tested cohort are in agreement with serum MDA levels previously found in human after several hours of fasting [29]. High MDA levels were defined above the sample median corresponding to 12.87, 12.10, and 13.46 (nM) among the entire sample, men and women, respectively.

### 2.5. Statistical Analysis

Statistical analyses were performed using SPSS version 25 (IBM-SPSS Armonk, NY) software. Continuous variables are presented as means ± SD. To test differences in continuous variables between two groups, the independent samples t-test was performed. Associations between nominal variables were performed with the Pearson Chi-Square test, and P for trend was calculated (Linear-by-Linear association of Chi-Square test) when needed. A multivariate logistic regression analysis was performed to test the adjusted association between MDA and NAFLD, NASH or fibrosis, or between vitamin E or C and MDA, adjusting for potential confounders (relevant variables which were different between the levels of serum MDA as depicted in Table 1). Odds ratio (OR) and 95% confidence interval (CI) are presented. *P* value of <0.05 was considered statistically significant for all analyse.

## 3. Results

### 3.1. Description of the Study Population and Comparison between Subjects with High (by Median) and Low MDA

**Figure 1:**
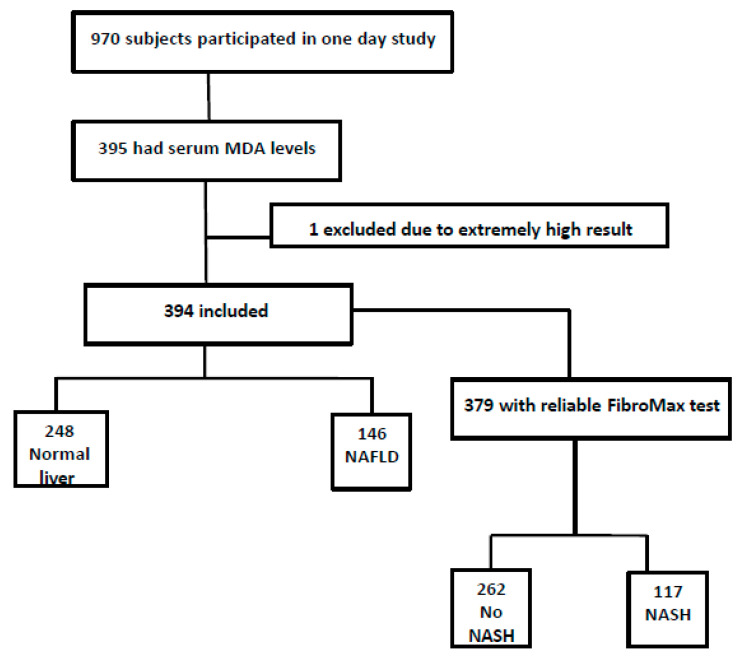
Flow chart of study population.

Out of 970 subjects who participated in the study, 789 were eligible as previously described [30]. From those, 395 had available serum samples for the serum MDA measurements (one sample was excluded due to extreme result), 53% were men, mean age was 59.15 ± 6.45 years and mean body mass index (BMI) was 28.35 ± 5.51 Kg/m^2^. NAFLD was diagnosed by AUS in 37% (n = 146). Reliable FibroMax test was obtained from 379 subjects (15 had no serum sample). Presumed NASH was observed in 30.9% (n = 117) and presumed significant fibrosis in 6.3% (n = 24) (Figure 1).

**Figure A1:**
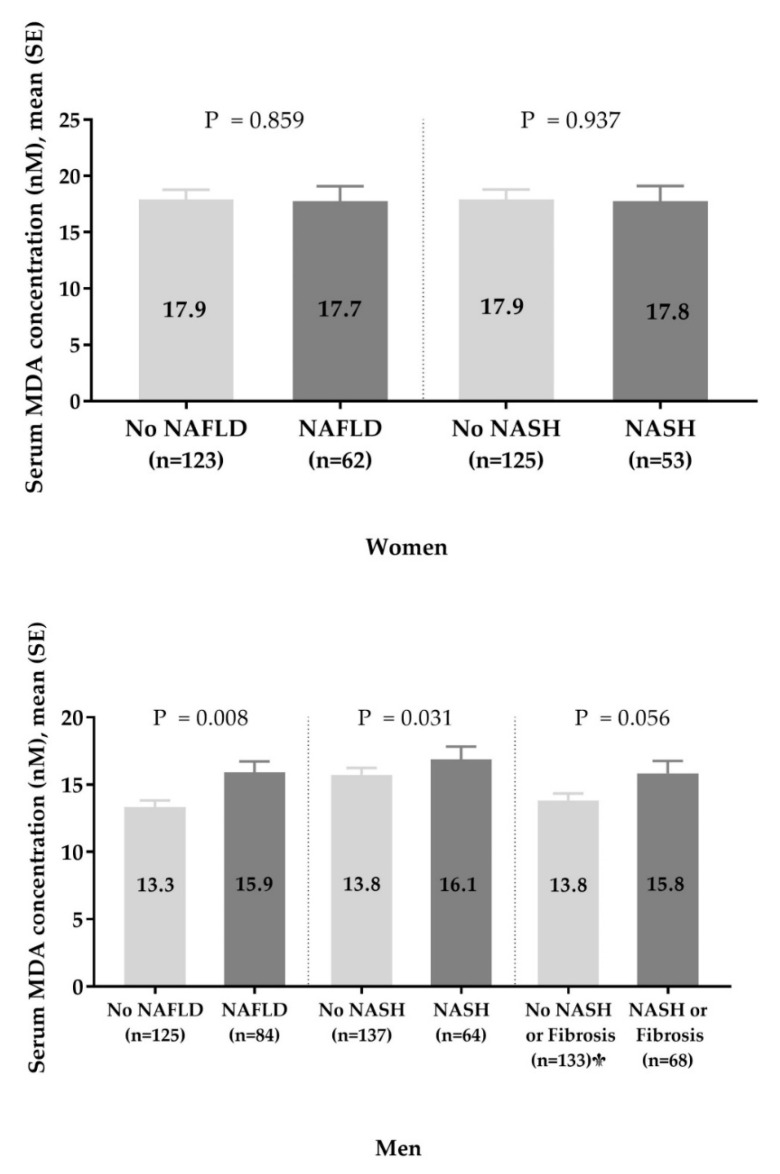
Mean serum MDA levels [±standard error (SE)] across non-alcoholic fatty liver disease (NAFLD) and steatohepatitis (NASH) diagnosis, stratified by gender. (Malondialdehyde, MDA Note: Among women, there were no cases of significant fibrosis that were without NASH, thus no results are presented in this category.)

There was a significant difference of serum MDA levels between men and woman (14.37 ± 6.50 vs. 17.89 ± 9.79 nM, *p* < 0.001, respectively), and we stratified the results by gender to explore potential modifying effect of gender. Indeed, mean serum MDA levels [±standard error (SE)] were higher among men with NAFLD vs. those without and among men with presumed NASH vs. those without, but no differences were noted among women (Figure A1).

Women with higher serum MDA levels had lower BMI and waist circumference. Men with higher serum MDA levels had higher levels of fasting glucose and HbAI1C (%), higher ALT levels and NASH-test score, consumed significantly more calories per day and saturated fatty acids (SFA) as percent of total calories. Both women and men with higher serum MDA levels had higher aspartate transaminase (AST) levels, consumed significantly less sugared beverages and vitamin E (per 1000 daily calories), but more cups of coffee (Table 1). The variables that differed between subjects with low and high MDA levels were considered as potential confounders in the multivariable analysis.

### 3.2. Dose-Response Association of MDA Levels and NAFLD among the Entire Study Sample and by Gender

**Figure 2:**
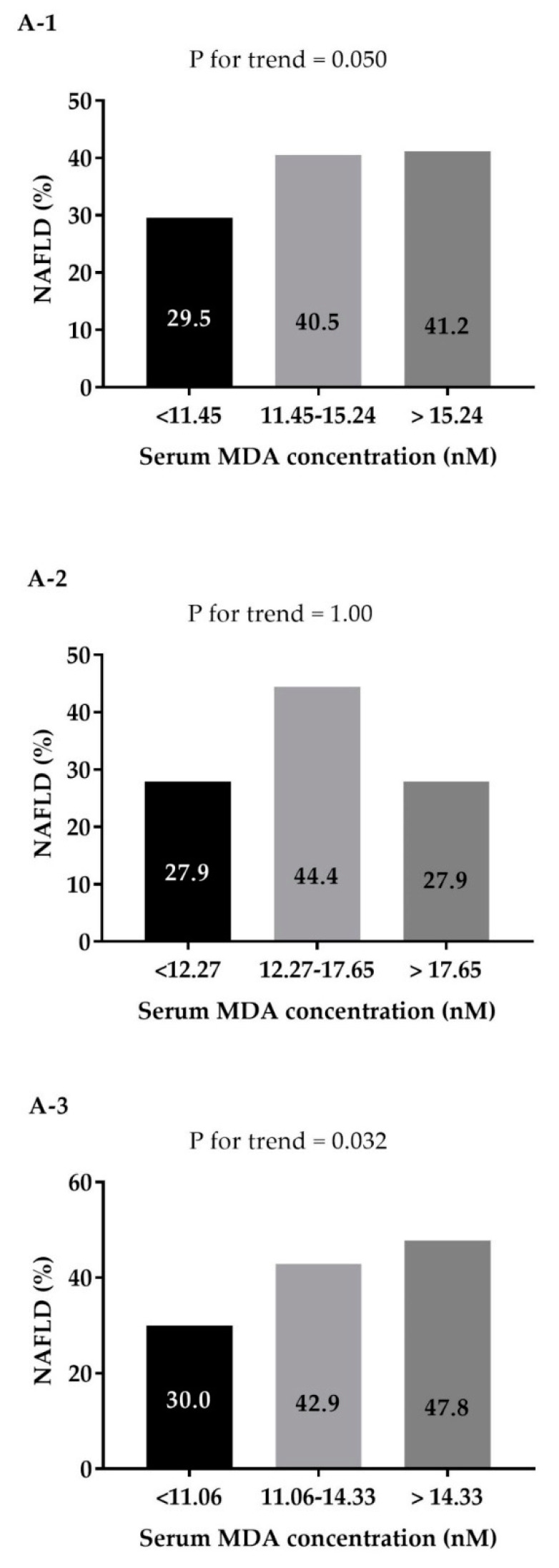

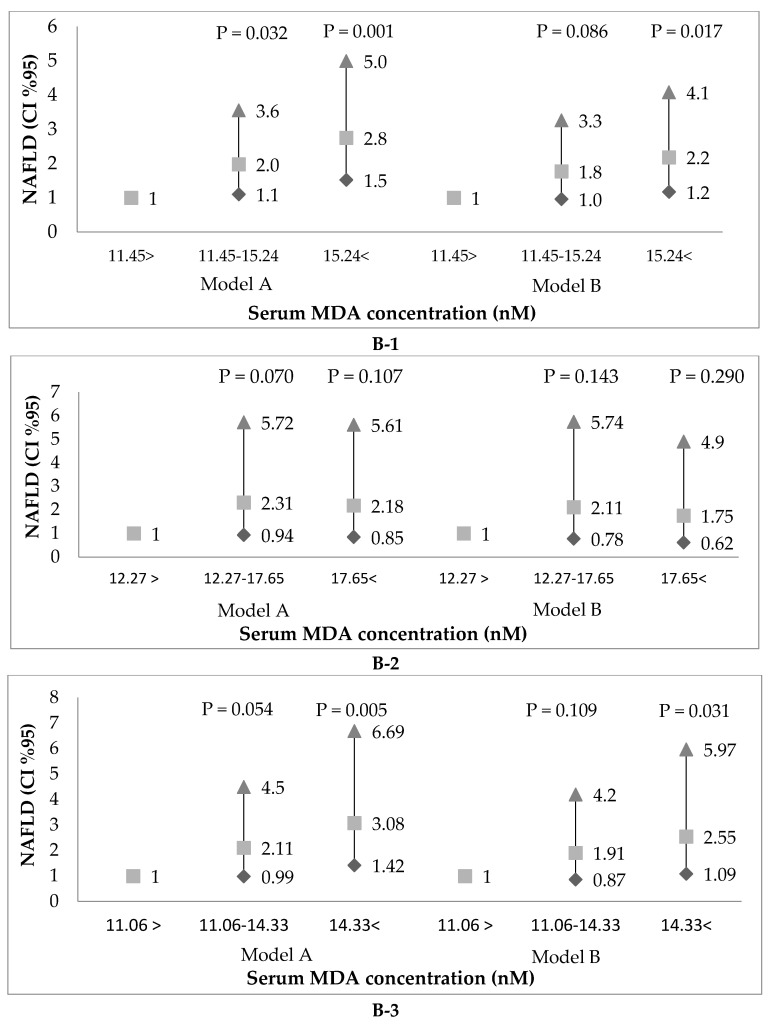
Univariate (**A**) and multivariate (**B**) dose response association between serum MDA concentration (nM) (tertiles, entire sample and gender specific) and NAFLD among the entire sample (**A-1** and **B-1**), women (**A-2** and **B-2**), and men (**A-3** and **B-3**). In multivariate analysis Model^A^ adjusted for: age (years), gender (in analysis of the entire sample), BMI (Kg/m^2^) and energy intake (Kcal/d). Model^B^ additionally adjusted for: pack years, physical activity (h/w) SFA (% total Kcal) coffee (portions/d), total sugared beverages (portions/d) and A1C (%).

In a univariate analysis, the prevalence of NAFLD was higher across increased levels of serum MDA in a dose–response manner (Figure 2(A-1)). In a multivariate analysis the odds for NAFLD were significantly higher with the rise of serum MDA levels in a dose–response manner, adjusting for age, gender, BMI and energy intake (Figure 2(B-1), model A), and with further adjustment for additional lifestyle habits (Figure 2(B-1), model B). Stratification of this analysis by gender, revealed a dose–response association among men, but not among women (Figure 2(B-3,B-2) respectively).

### 3.3. Multivariate Association of Serum MDA Levels and NAFLD and Presumed Related Liver Damage Stratified by Gender

In a multivariate analysis, adjusting for age (years), BMI (Kg/m^2^), energy (Kcal/day), gender, pack years, physical activity (h/w) SFA (% total Kcal) coffee (portions/d), total sugared beverages (portions/d) and A1C (%), the upper median of serum MDA levels was associated with NAFLD among the entire sample (OR = 1.93, 95% CI 1.15–3.24, *P* = 0.013). In addition, among men, the upper median of serum MDA was associated of higher odds for NAFLD and NASH and/or fibrosis (OR = 2.59, 95% CI 1.33–5.07, *P* = 0.005; OR = 2.04, 1.02–4.06, *P* = 0.043, respectively). There was no association between serum MDA and NAFLD or liver damage among women (Table 2).

### 3.4. Multivariate Association of Vitamins E and C Intake and Serum MDA Levels Stratified by Gender 

In a multivariate analysis, adjusting for age, gender, BMI, pack years, physical activity, SFA, coffee, total sugared beverages and A1C, the upper tertile of vitamin E intake (mg/1000 Kcal) was associated with lower odds of high serum MDA levels in the entire population and among women (OR = 0.28 95% CI 0.13–0.62, *P* = 0.002; OR = 0.27, 0.08–0.92, *P* = 0.036, respectively). There was a similar tendency among men, but it did not reach statistical significance. No association was observed with vitamin C (Table 3).

## 4. Discussion

Previously it was reported in small-scale studies that NASH patients are susceptible to increased levels of circulating lipid peroxidation products. Patients with NASH had higher levels of lipid peroxidation end-products with correlation to increase in cardiovascular risk [31]. Here we are demonstrating by evaluation of a large sample of people from a general population, that there is a strong independent association between NAFLD, presumed NASH and circulating levels of oxidized lipids. Interestingly, there was an interaction with gender, where the association was evident only in men. Our results also indicate strong inverse association of MDA levels and consumption of vitamin E, which may indicate lower antioxidant status.

There are only few epidemiological studies testing the independent association between serum MDA levels and NAFLD, none of them thoroughly adjusted for lifestyle risk factors or tested gender differences. In a study including 139 individuals with type-2 diabetes, MDA was an independent predictor of higher fatty liver index (FLI) as a marker for NAFLD [32]. Similarly, a study conducted on 300 patients with type-2 diabetes, MDA was elevated in AUS diagnosed NAFLD patients [33]. An association between MDA levels and disease severity has been demonstrated in small sample studies with liver biopsy. In a small study including 32 patients with biopsy-proven NAFLD, both serum MDA and tissue MDA were independently associated with increased homeostatic model assessment for insulin resistance (HOMA-IR), and increased MDA was a risk factor for NASH [34]. Similar findings were demonstrated in 67 patients with NAFLD or NASH, where serum MDA was significantly elevated in NAFLD and NASH groups compared to controls [35]. Considering the fact that NAFLD and NASH improve with weight reduction [36], another support for our findings is the reduction in MDA levels following lifestyle or bariatric interventions. Elevations in hepatic lipid peroxidation improve significantly with weight loss induced by bariatric surgery, as indicated by liver biopsies from 20 NAFLD patients [37]. Similarly, dietary intervention followed by weight reduction led to reduction in serum MDA levels [38]. Specifically, the Dietary Approaches to Stop Hypertension (DASH) diet, that is abundant with antioxidants, designed to be rich in fruits, vegetables, and whole grains, led to reduction in serum levels of inflammatory markers including MDA among patients with NAFLD [39].

In animal models, the severity of NASH was correlated to ferroptosis (death of cells by lipid peroxidation). Elevated arachidonic acid metabolism was reported to promote ferroptosis in methionine-choline deficient diet fed mouse livers, which was further demonstrated by lipid ROS accumulation, morphological change of mitochondria and increased cell death [40]. Age-related fibrosis in NASH patients was also connected to changes in redox cellular status. Fibrosis was demonstrated to be modulated by p52Shc/NOX2 that results in redox stress, and accelerated fibrosis in the aged due to increased production of hydrogen peroxides and superoxides that can promote lipid peroxidation as was demonstrated in old mice [41].

The reason why women are more protected against hepatic production of oxidized lipids could be related to the effect of estrogen. Indeed, direct evidence can be observed by treatment with 17beta-estradiol in aging male rats. Such treatment has a protective effect and prevented increase in lipid peroxidation, hepatic dysfunction and histological changes [42]. Another study demonstrated that ovariectomy in female rat can cause lipid peroxidation in liver tissues, while estradiol and progesterone supplementations to the ovariectomized rats protect against lipid peroxidation to a large extent [43].

In this study, we found an inverse association between dietary vitamin E intake and serum MDA levels among women, similar tendency was noted in men but it did not reach statistical significance. No similar association was noted with vitamin C. We have previously found in the same study population, that vitamin E and vitamin C intake were associated with lower odds of NAFLD and NASH [44]. These finding imply that the protective effect of vitamin E, as a fat-soluble antioxidant, may be mediated, at least partially, by reduction of MDA levels among women. Conversely, vitamin C, as a water soluble vitamin, may act through different mechanisms, but, of course, no causal inference can be determined in a cross-sectional study. Indeed, vitamin E consumption has been demonstrated among humans, to have a favorable effect in reduction of NASH, but not fibrosis [45,46]. However, while the recommended intake of vitamin E for adults is 15 mg/day (or 22.4 IU/day) [47], the supplements studies contained 400–800 IU. Since high dose vitamin E supplement raises some concerns regarding increased risk for mortality [48], hemorrhagic stroke [49] and prostate cancer [50], testing a potential protective effect of a lower dose provided by diet is appealing. Vitamin E is one of the most powerful chain-breaking antioxidants in the human body and has the ability to repress peroxidation and inhibit the expression of transforming growth factor-beta, which has been associated with hepatic fibrosis and hepatocyte apoptosis by activating hepatic stellate cells. By preventing the nuclear localization of NF-κB, decreasing COX-2 expression, and suppressing the expression of cytokines, vitamin E is able to dampen the inflammatory response in NAFLD [51]. In addition, immuno-histological evaluation demonstrated that vitamin E modulation decreases the Hedgehog response in the liver, which is elevated in chronic liver injury and promotes fibrosis and inflammation [52].

The best sources of vitamin E are nuts, seeds, plant oils, green leafy vegetables, and fortified cereals [47]. Therefore, it may be that higher vitamin E intake is an indicator for a better diet. To control for that, we adjusted the association for caloric intake as well as for major dietary components and other lifestyle behaviors as smoking and physical activity, and the association with MDA persisted. The fact that there was an association only with vitamin E and not with vitamin C intake, which is also a marker for a better nutrition in general, strengthen the assumption that vitamin E has a specific association with lower MDA levels.

The strengths of the study include its large sample size and meticulous assessment of dietary intake as well as other lifestyle parameters that could be controlled for. We were also able to reveal gender differences. However, this study has several limitations to consider. First, the cross-sectional design of the study does not allow causal inference. Second, dietary habits were self-reported and thus prone to a report bias. However, since the participants and the research team were both blinded to the AUS and FibroMax results, it is a non-differential repot bias and therefore may have only weakened the observed associations. Third, the diagnosis of NAFLD and presumed NASH or fibrosis were determined by AUS and FibroMax markers, respectively, and not by liver histology, which cannot be performed in a sample of apparently healthy volunteers. However, AUS it is the most accepted and common screening method for NAFLD in a general population. The FibroTest validity has been demonstrated in several studies and biomarkers of fibrosis are considered as reasonably acceptable non-invasive procedures, but as for the reference NashTest, although validated, non-invasive tests are still considered to be insufficient for the diagnosis of NASH.

## 5. Conclusions

Serum MDA is strongly associated with NAFLD and markers of NASH or fibrosis among men. Dietary vitamin E intake seems to be protective from elevated MDA levels among women, suggesting a pathway for its protective role in NAFLD and mainly NASH. Prospective studies are needed to confirm these findings.

## Figures and Tables

**Table 1:** Comparison between subjects with high and low malondialdehyde (MDA) (by gender specific median, nM) (Mean ± SD, unless otherwise stated).

Variable (Units, Normal Range)	Women			Men		
	MDA < 13.46 (n = 92)	MDA ≥ 13.46 (n = 93)	*p* Value	MDA < 12.10 (n = 104)	MDA ≥ 12.10 (n = 105)	*p* Value
**Age (years)**	58.50 ± 6.92	59.73 ± 6.26	0.206	59.06 ± 6.59	59.30 ± 6.09	0.787
**BMI (Kg/m^2^)**	28.76 ± 6.20	26.53 ± 4.43	0.006	29.45 ± 6.19	28.50 ± 4.65	0.214
**Waist Circumference (cm)**	96.27 ± 14.55	92.07 ± 11.95	0.033	105.27 ± 12.57	103.42 ± 13.50	0.309
**HOMA-IR (score)**	2.61 ± 1.86	2.49 ± 2.10	0.682	3.05 ± 2.14	3.04 ± 1.89	0.990
**HbA1C (%)**	5.82 ± 0.50	5.85 ± 0.61	0.671	5.77 ± 0.56	6.10 ± 1.01	0.004
**Triglyceride (mg/dl)**	101.73 ± 48.39	107.44 ± 53.15	0.446	122.70 ± 81.71	116.04 ± 76.63	0.544
**Total Cholesterol (mg/dl)**	186.13 ± 33.18	192.48 ± 38.62	0.232	167.73 ± 30.66	171.30 ± 36.14	0.443
**ALT (U/L, 5-39)**	22.70 ± 13.51	24.38 ± 10.08	0.338	23.92 ± 11.29	27.54 ± 11.65	0.024
**AST (U/L, 7-40)**	22.81 ± 11.16	26.19 ± 10.30	0.033	22.38 ± 6.15	25.10 ± 7.00	0.003
**GGT (U/L, 6-28)**	29.85 ± 37.65	25.25 ± 27.62	0.345	27.91 ± 18.18	29.50 ± 28.96	0.637
**NashTest (score)**	0.40 ± 0.17	0.45 ± 0.15	0.022	0.42 ± 0.12	0.48 ± 0.13	0.001
**NASH (%)**	28.10	31.50	0.623	23.20	40.20	0.010
**FibroTest (score)**	0.16 ± 0.12	0.14 ± 0.11	0.291	0.25 ± 0.16	0.27 ± 0.17	0.405
**Significant Fibrosis (%)**	2.20	2.20	1.00	7.10	12.70	0.179
**Uric Acid (mg/dl, 2.3-6)**	4.88 ± 1.20	4.87 ± 1.23	0.982	5.93 ± 1.23	6.07 ± 1.19	0.401
**C-Reactive Protein (mg/L, <5)**	4.15 ± 4.91	3.99 ± 5.43	0.837	4.10 ± 7.63	3.71 ± 6.02	0.676
*Nutritional and lifestyle habits*						
**Energy (Kcal)**	2153.82 ± 702.57	1971. 84 ± 644.25	0.068	2253. 95 ± 740.16	2031. 67 ± 712.68	0.028
**Saturate Fatty Acid (% of total Kcal/d)**	12.48 ± 3.97	12.34 ± 4.68	0.827	11.49 ± 3.24	12.54 ± 3.93	0.036
**Cholesterol (mg/d)**	337.28 ± 225.02	290.57 ± 131.96	0.088	367.35 ± 195.32	364.40 ± 239.01	0.922
**Fiber (gr/d)**	23.95 ± 12.98	23.17 ± 10.86	0.659	23.95 ± 13.07	21.60 ± 12.45	0.184
**Red/Processed Meat (portions/d)**	0.40 ± 0.66	0.33 ± 0.44	0.428	0.90 ± 1.13	0.76 ± 1.01	0.340
**Total Fish (portions/d)**	0.42 ± 0.48	0.55 ± 0.68	0.151	0.51 ± 0.46	0.58 ± 0.54	0.332
**Sugared Beverages** **(cups/d)**	3.71 ± 4.09	2.11 ± 3.61	0.005	3.87 ± 3.96	2.02 ± 3.31	< 0.001
**Coffee (cups/d)**	2.56 ± 2.82	3.49 ± 3.46	0.048	2.41 ± 2.71	3.48 ± 3.47	0.014
**Alcohol (portions/d)**	0.73 ± 1.79	0.76 ± 1.51	0.902	2.55 ± 3.63	2.42 ± 3.53	0.785
**Vitamin E (mg/ 1000 Kcal)**	9.65 ± 6.77	7.53 ± 6.64	0.033	9.28 ± 6.67	6.16 ± 5.44	< 0.001
**Vitamin C (mg/ 1000 Kcal)**	89.41 ± 58.54	91.78 ± 71.94	0.806	92.20 ± 61.17	79.01 ± 46.51	0.081
Pack Years *	11.23 ± 18.66	12.19 ± 23.42	0.758	19.86 ± 25.24	18.68 ± 25.30	0.735
**Physical Activity (hours/week)**	1.60 ± 2.66	2.37 ± 3.21	0.076	1.90 ± 3.52	2.76 ± 3.54	0.079

Malondialdehyde, MDA; body mass index, BMI; homeostatic model assessment for insulin resistance, HOMA-IR; Alanine aminotransferase, ALT; aspartate transaminase, AST; Gamma-glutamyl transferase, GGT. * Pack years were defined as cigarettes per day divided by 20 cigarettes per pack, multiplied by years of smoking. Never smokers were considered as zero.

**Table 2:** Multivariate association of serum MDA concentration (nM) and presumed related liver damage.

	NAFLD	NASH	Fibrosis ≥ F2 or NASH
	OR (95% CI), P		
All Samples (n Cases) ^a^	146	117	121
**Model A**			
<12.87	1 (ref)	1 (ref)	1 (ref)
≥12.87	2.24 (1.38–3.64), 0.001	1.51 (0.93–2.44), 0.094	1.46 (0.91–2.36). 0.121
**Model B**			
<12.87	1 (ref)	1 (ref)	1 (ref)
≥12.87	1.93 (1.15–3.24), 0.013	1.14 (0.69–1.90), 0.612	1.12 (0.67–1.86), 0.666
**Women** **(n Cases)**	**62**	**53**	**53**
**Model A**			
<13.46	1 (ref)	1 (ref)	1 (ref)
≥13.46	1.69 (0.79–3.63), 0.174	1.10 (0.53–2.31), 0.794	b
**Model B**			
<13.46	1 (ref)	1 (ref)	1 (ref)
≥13.46	1.37 (0.60–3.14), 0.461	0.84 (0.37–1.87), 0.661	b
**Men** **(n Cases)**	**84**	**64**	**68**
**Model A**			
<12.10	1 (ref)	1 (ref)	1 (ref)
≥12.10	2.87 (1.53–5.37), 0.001	2.20 (1.16–4.21), 0.017	2.22 (1.17–4.21), 0.015
**Model B**			
<12.10	1 (ref)	1 (ref)	1 (ref)
≥12.10	2.59 (1.33–5.07), 0.005	1.95 (0.98–3.91), 0.059	2.04 (1.02–4.06), 0.043

Malondialdehyde, MDA; non-alcoholic fatty liver, NAFLD; Non-alcoholic steatohepatitis, NASH. Model A adjusted for: age (years), BMI (Kg/m^2^) and energy (Kcal/day). Model B additionally adjusted for: pack years, physical activity (h/w) SFA (% total Kcal) coffee (portions/d), total sugared beverages (portions/d) and A1C (%). Notes: ^a^ Additionally adjusted for gender. ^b^ Among women, there were no cases of significant fibrosis that were without NASH, thus no results are presented in this category.

**Table 3:** Multivariate association of vitamins E and C dietary intake and high MDA levels (above the sample median).

**Variable**	All Sample ^a^		Women		Men	
	Value	OR (95% CI) P	Value	OR (95% CI) P	Value	OR (95% CI) P
**Vitamin E (>upper tertile, mg/1000 Kcal)**	<8.43	1 (ref)	<8.40	1 (ref)	<8.48	1 (ref)
	≥8.43	0.28 (0.13–0.62) 0.002	≥8.40	0.27 (0.08–0.92) 0.036	≥8.48	0.37 (0.13–1.08) 0.068
**Vitamin C (>upper tertile, mg/1000 Kcal) intake**	<97.83	1 (ref)	<102.84	1 (ref)	<94.93	1 (ref)
	≥97.83	1.12 (0.63–1.97) 0.707	≥102.84	0.88 (0.39–2.00) 0.766	≥94.93	1.03 (0.47–2.24) 0.947

Malondialdehyde, MDA. All models are adjusted for: age (years), BMI (Kg/m^2^), pack years, physical activity (h/w) SFA (% total Kcal) coffee (portions/d), total sugared beverages (portions/d) and A1C (%).^a^ additionaly adjusted for gender.
